# Topiramate modulates habituation in migraine: evidences from nociceptive responses elicited by laser evoked potentials

**DOI:** 10.1186/1129-2377-14-25

**Published:** 2013-03-12

**Authors:** Laura Di Clemente, Francesca Puledda, Antonella Biasiotta, Alessandro Viganò, Edoardo Vicenzini, Andrea Truini, Giorgio Cruccu, Vittorio Di Piero

**Affiliations:** 1 Department of Neuroscience, Sapienza University of Rome, viale dell’Università 30, Rome 00185, Italy

**Keywords:** Laser evoked potentials, Habituation, Preventive treatment, Topiramate

## Abstract

**Background:**

Lack of habituation during repetitive stimulation is the most consistent interictal abnormality of cortical information processing observed in migraine. Preventive migraine treatments might act by stabilizing cortical excitability level and thus the habituation to external stimuli.

**Methods:**

We examined the effects of preventive treatment with topiramate on migraineur’s habituation to nociceptive stimulation. Scalp potentials were evoked by Nd-YAP Laser stimulation of the hand dorsum and supraorbital region in 13 patients with migraine without aura (MO) and 15 healthy volunteers (HV). The exam was repeated in MO before and after treatment.

**Results:**

We observed a lack of habituation and lower initial amplitudes in MO compared to HV. These abnormalities reached statistical significance for N1 LEPs component, generated in the secondary somatosensory cortex (SII), but not for N2/P2 complex, generated in the insula and anterior cingulated cortex (ACC). Topiramate normalized the N1 habituation pattern in MO, with a significant correlation between clinical effects and normalization of neurophysiological responses.

**Conclusions:**

Our results indicate a modulating action of topiramate on cortical processing of sensorial stimuli, mainly regarding the sensory-discriminative component of pain, elaborated by SII, without a significant effect on the affective dimension of pain, in which the ACC has an important role.

## Background

The complex mechanism that underlies migraine pathology can be investigated in patients through the methods of clinical neurophysiology. Lack of habituation responses during repetitive stimulation is the most consistent interictal abnormality of cortical information processing observed. This has been described for every sensory modality of stimulation using different evoked potentials [1,2]. Laser evoked potentials (LEPs) have confirmed this cortical dysfunction also in pain elaboration [3,4]. Laser stimulation activates selectively Aδ and C fibres generating an evoked potential that can be recorded from the temporal regions (early components) and the vertex (late components) of the skull [5-7]. Evidences from dipolar analysis suggest that the early component is mainly generated in the secondary somatosensory cortex, while the late component in the insular and anterior cingulate cortex [8]. Laser evoked responses are thus particularly interesting for the study of migraine because they allow to distinguish between two components of pain: sensory-discriminative and affective-motivational.

In addition, LEP and blink reflex recordings have shown similar habituation patterns in migraineurs after stimulation of trigeminal and extra-trigeminal areas. This suggests that the habituation deficit found in migraineurs may be due to an altered central processing of sensorial information, and not to peripheral sensitization [9,10]. This is probably due to abnormal monoaminergic control of cortical responsiveness and altered neuronal excitability [11,12].

It has been hypothesized that this abnormal pattern might favour the trigger and the repetition of migraine attacks [13], and therefore it could represent a possible target for migraine prophylaxis.

We still own a poor knowledge of how migraine preventive treatments act, and this is particularly true for those drugs more recently introduced in migraine therapy. A recent paper showed that the deficient LEP habituation in medication overuse headache is partly restored after detoxification and administration of several preventive drugs (flunarizine, topiramate, amitriptyline, gabapentin, cinnarizine and levetiracetam), in patients who improved after therapy [14]. Several studies have shown modulation of habituation deficit by beta-blockers [15,16], but still few neurophysiological data exist on the mechanisms through which antiepileptic drugs prevent migraine.

In this regard, De Tommaso et al. observed that both topiramate and levetiracetam are able to reverse the abnormal CNV habituation index in migraine patients, with a correlation between the reduction of migraine frequency and the habituation index after treatment [17]. Topiramate blocks voltage-dependent Na-channels, potentiates GABA activity, inhibits AMPA/KA receptors thus reducing excitatory glutamate transmission, and blocks L- and N-calcium channels [18]. These biological effects are probably responsible for the inhibition of cortical spreading depression (CSD) by topiramate observed in animal migraine models [19]. It has been hypothesized that antiepileptic drugs might protect migraineur’s brain from attacks by stabilizing cortical excitability level and thus the response to external nociceptive stimuli [20].

For the above-described reasons, we investigated if topiramate could also act on migrainous brain vulnerability by restoring the physiological habituation pattern of nociceptive processing, and we verified if this action is more evident in those patients that show a better response to treatment.

## Methods

### Subjects

We enrolled 22 patients with a diagnosis of migraine without aura (MO, code 1.1) according to ICHD-II criteria (Headache Classification Subcommittee of the International Headache Society) with episodic pattern of 3 or more attacks/month [21]. They were compared with 15 healthy volounteers (HV). All subjects were devoid of any other pathology than migraine, and HV had no personal or family history of recurrent headache. Migraine patients had never taken any prophylactic treatment before recruitment.

We used a clinical diary to check headache characteristics (quality, intensity and site of pain, accompanying symptoms), as well as attack frequency and duration. We used a 10-point Numeric Rating Scale (NRS) to measure pain intensity, considering 10 as intolerable pain and 0 as no sensation. We selected patients eligible for the study after a 1-month baseline diary. Once enrolled, migraineurs began preventive treatment with topiramate after the first recording session.

Written informed consent was obtained from all participants in accordance with the Declaration of Helsinki and the study protocol was approved by the local Ethics Committee.

### Data acquisition

We studied the cortical pain processing after nociceptive stimulation in trigeminal and extra-trigeminal areas, applying YAP laser stimuli.

One LEPs recording session for each healthy volounteer and two recording sessions for each patient were applied: before (T0) and after at least 2 months of preventive treatment with topiramate (T1). In MO group, LEPs were recorded interictally, at least 2 days after the last and before the next migraine attack. The clinical outcome was evaluated using the same monthly headache diary, before (baseline diary) and during treatment.

Laser stimulations were applied on the right supraorbital region (V1) and the right hand dorsum. We decided to stimulate unilateral areas to avoid a lengthy procedure, as no patients presented fixed unilateral migraine. The sensory and painful thresholds were determined on both regions by the method of limits in three series of increasing and decreasing stimulus intensities. In the LEPs recording sessions, we used a fixed intensity set at 2.5 times the individual sensory threshold, defined as the lower stimulus intensity that elicited a distinct painful pinprick sensation. Subjects defined recording intensity distinctly for face and hand by using the NRS scale.

All subjects underwent a standard recording session with three scalp electrodes placed in the vertex and the temporal region contralateral to the stimulation side (respectively Cz and T3 positions of the 10–20 International System). The reference electrode was placed in the forehead (Fz) and the ground on the wrist. During all the recordings, we monitorized eye movements by electro-oculography.

The sequence of the stimulation sites varied randomly across subjects. Three consecutive blocks of responses were obtained for each stimulation site. Each block consisted of 15 sweeps, with an inter-stimulus interval of 10 seconds, and an interblock interval of 5 minutes. The responses obtained for each stimulation block were filtered (bandpass: 1 Hz-2 KHz) and averaged off-line.

### Data processing and statistical analysis

LEPs recordings were analyzed by investigators, blinded of the subjects’ clinical conditions.

On each block average, we measured the latency and the peak-to-peak amplitude of the temporal (N1) and vertex (N2/P2) component. Habituation was calculated measuring amplitude percentage changes between the first and last block of responses. Negative percentage values therefore indicate habituation, i.e. reduction of amplitude, whereas positive percentage values reflect potentiation, i.e. amplitude increase across the repetition.

We analysed differences between HV and MO groups and in the latter before and during treatment with regards to pain and sensory thresholds, LEPs amplitude, latency and habituation. After performing Shapiro-Wilk’s test to assess normality, group differences in all collected data at baseline were calculated with Mann–Whitney test, while data changes in MO group between T0 and T1 recording sessions were analyzed with Wilcoxon test. When necessary, post hoc analysis was performed using Bonferroni’s correction.

In migraine patients, we also correlated habituation changes and clinical improvement in terms of attack frequency reduction. Spearman’s correlation was used to search for intraindividual correlation.

Results were considered significant at p ≤ 0.05.

## Results

Out of 22 enrolled patients, 9 did not finish the study (1 for collateral effects caused by topiramate, 2 for lack of compliance to therapy, 4 because lost before the T0 LEP session and 2 because lost to T1 LEP session). We included the remaining 13 MO patients (4 male, 9 female, mean age: 38.5 ± 12.0) comparing them with 15 HV (5 male, 10 female, mean age: 30.9 ± 5.7).

Migraine patients had a mean headache frequency of 5.8 ± 2.2 attacks/months at baseline with a mean headache intensity of 8.2 ± 1.0 (NRS value). Depending on patient’s tolerance, the mean daily dose of topiramate was 75 mg (range 50–100 mg). Patients underwent T1 LEP recording session after 63.7 ± 4.9 days of treatment.

We observed a significant clinical improvement at T1 in all patients. They presented a mean headache frequency of 1.9 ± 1.1 attacks/months (−65%, p < 0.001), with a mean headache intensity of 6.6 ± 1.6 (−20%, p = 0.014).

We found no significant differences in perceptive and pain thresholds and thus in stimulation intensity between HV and MO group, and in the latter between T0 and T1. In all recordings, LEPs components were clearly identified.

The latencies of both N1 and N2/P2 complex measured after supraorbital and hand stimulations were shorter in patients than in controls, but this difference did not reach statistical significance (Table 1).

**Table 1:** Latencies (msec) and first block amplitudes (μV) of the LEPs complex detected after V1 and hand stimulation (mean ± standard deviation)

	**MO (T0)**	**MO (T1)**	**HV**	
N1 latency, V1	133.7 ± 14.4	129.4 ± 13.4	138.9 ± 11.9	
N2/P2 latency, V1	172.5 ± 10.0	166.3 ± 7.1	175.9 ± 16.7	
N1 latency, hand	162.7 ± 22.2	161.8 ± 20.8	172.9 ± 22.5	
N2/P2 latency, hand	200.5 ± 16.1	199.8 ± 16.4	212.1 ± 19.5	
N1 amplitude, V1	5.2 ± 4.1	8.7 ± 4.2	10.0 ± 4.1	MO (T0) vs HV, p = 0.0047 (p = 0.009*)
				MO (T0) vs MO (T1), p = 0.03
				MO (T1) vs HV, NS (p = 0.26)
N2/P2 amplitude, V1	31.2 ± 18.5	34.1 ± 13.0	38.2 ± 9.3	MO (T0) vs HV, p = 0.0043 (p = 0.008*)
				MO (T0) vs MO (T1), NS (p = 0.34)
				MO (T1) vs HV, NS (p = 0.29)
N1 amplitude, hand	6.1 ± 4.7	6.3 ± 5.3	10.3 ± 5.1	MO (T0) vs HV, p = 0.018 (p = 0.036*)
				MO (T0) vs MO (T1), NS (p = 0.75)
				MO (T1) vs HV, p = 0.014 (p = 0.028*)
N2/P2 amplitude, hand	33.3 ± 17.6	34.7 ± 14.2	42.2 ± 18.0	MO (T0) vs HV, NS (p = 0.14)
				MO (T0) vs MO (T1), NS (p = 0.70)
				MO (T1) vs HV, NS (p = 0.34)

Statistical significance after Bonferroni correction is marked by *.

Latencies did not change in any subject group across the stimuli repetition.

**Figure 1:**
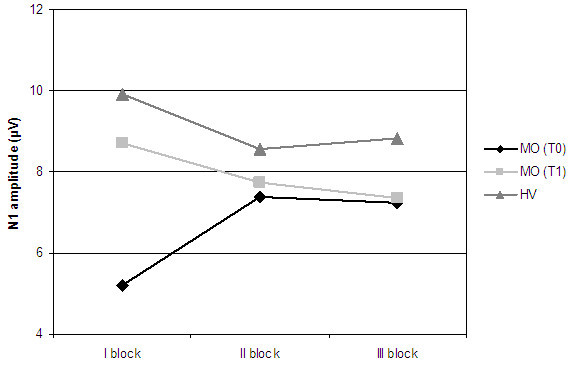
N1 block amplitudes obtained after stimulation of V1 district.

**Figure 2:**
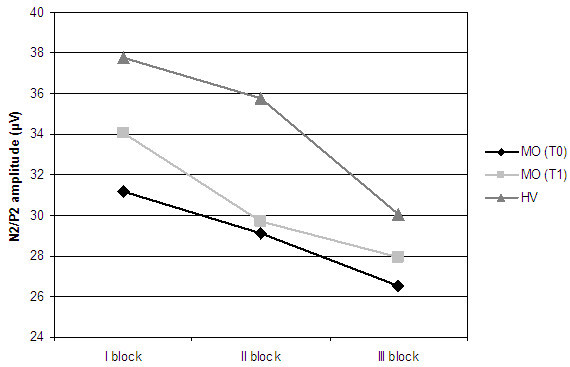
N2/P2 block amplitudes obtained after stimulation of V1 district.

The mean first block N1 and N2/P2 amplitudes were greater in HV respect to MO both in T0 and T1 recording sessions (Table 1, Figures 1 and 2). This difference at baseline was significant for N1 component after V1 and hand stimulation (p = 0.0047 and p = 0.018, respectively), and for N2/P2 component after V1 stimulation (p = 0.0043), with a tendency to significance for hand stimulation (p = 0.14). Furthermore, mean first block amplitudes of N1 responses evoked by supraorbital stimulation showed a significant increase in MO after treatment (p = 0.03, Figure 1), while the difference with HV didn’t result to be significant.

**Figure 3:**
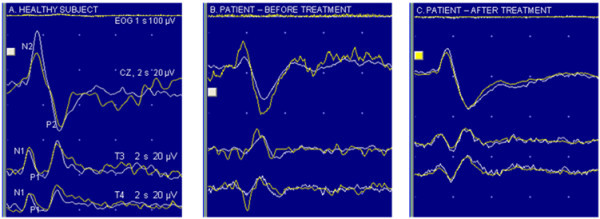
Laser evoked potentials after stimuli on left trigeminal (V1) area in one healthy subject (A) and in one patient (before and after treatment; B and C respectively); white lines (EOG, CZ, T3 and T4) show the average of the first 15 recordings, yellow lines show the average of the last 15 recordings.

We observed a clear deficit in habituation of N1 complex in patients at baseline respect to controls (Table 2, Figures 1 and 3). In the MO group we found an amplitude increase between the first and the last block of N1 evoked responses exceeding 90% after stimulation of V1 district and 16% after stimulation of the hand. This contrasted with the amplitude decrease (i.e. habituation) found in HV (−5.6% after V1 stimulation, p = 0.025, and −25.3% after hand stimulation, p = 0.015).

**Table 2:** Habituation of LEPs components expressed in percentage changes between the first and last block of evoked responses (mean ± standard deviation)

	**MO (T0)**	**MO (T1)**	**HV**	
V1: N1 habituation (%)	92.7 ± 45.9	−13.2 ± 38.1	−5.6 ± 27.5	MO (T0) vs HV, p = 0.025 (p = 0.05*)
				MO (T0) vs MO (T1), p = 0.039
				MO (T1) vs HV, NS (p = 0.39)
V1: N2/P2 habituation (%)	−16.4 ± 18.7	−18.3 ± 20.0	−18.7 ± 25.3	MO (T0) vs HV, NS (p = 0.8)
				MO (T0) vs MO (T1), NS (p = 0.7)
				MO (T1) vs HV, NS (p = 0.94)
Hand: N1 habituation (%)	16.9 ± 43.7	−2.9 ± 50.6	−25.3 ± 39.1	MO (T0) vs HV, p = 0.015 (p = 0.03*)
				MO (T0) vs MO (T1), NS (p = 0.46)
				MO (T1) vs HV, NS (p = 0.18)
Hand: N2/P2 habituation (%)	−18.8 ± 33.9	−31.6 ± 23.1	−23.9 ± 26.8	MO (T0) vs HV, NS (p = 0.69)
				MO (T0) vs MO (T1), NS (p = 0.22)
				MO (T1) vs HV, NS (p = 0.5)

After treatment, during T1 recording session, MO showed a reduction of the habituation deficit of N1 component, with an amplitude change of −13.2% after stimulation of V1 district, and −2.9% after stimulation of the hand. The difference in N1 habituation observed in MO group between T0 and T1 session reached statistical significance for responses recorded after supraorbital stimulation (p = 0.039).

The peak-to-peak amplitude of N2/P2 complex showed a minimum habituation deficit in patients at baseline respect to controls (Table 2, Figures 2 and 3). After stimulation of both districts, we observed a slight increase in habituation after therapy.

The subjective pain sensations defined by means of NRS values after laser stimulation tended to be lower in both districts in MO at baseline (5.3 ± 1.5 for V1; 5.5 ± 1.7 for hand) respect to HV (6.1 ± 1.4 for V1, 6.2 ± 0.8 for hand; p = 0.09, p = 0.1 respectively). We also observed a significant increase in NRS values in MO after treatment (6.3 ± 1.9 for V1, p = 0.05; 6.7 ± 1.5 for hand, p = 0.02).

**Figure 4:**
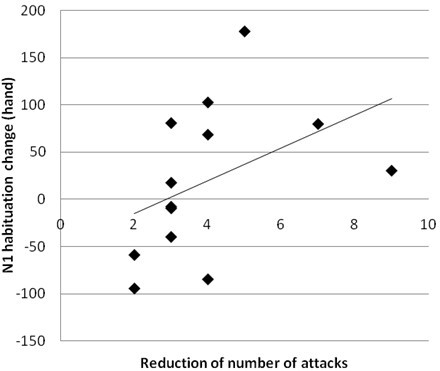
Correlation analysis between monthly attack frequency reduction and habituation change of N1 component after hand stimulation (p = 0.025, r = 0.62).

We found a positive correlation between clinical efficacy, expressed as attack frequency reduction, and the N1 habituation change after therapy in MO. This correlation was significant for N1 responses evoked after hand stimulation (p = 0.025, r = 0.62, Figure 4) and showed a tendency to significance after V1 stimulation (p = 0.09, r = 0.48).

## Discussion

Our study confirms the lack of habituation in MO patients [1,2] and the clinical efficacy of topiramate that resulted associated with a change in the habituation pattern.

The habituation deficit was greater for the early (N1) LEPs component that we found increased in amplitude after repetitive stimulation in MO, both for face and hand stimulation. We did not observe the same pronounced difference between patients and controls for the late (N2/P2) LEP component that showed only a slightly reduced habituation.

Previous results are consistent with the lack of habituation in migraine patients, despite the application of different experimental paradigms and data analysis produced dissimilar results [3,4].

The analysis of both LEP’s components allows the study of different aspects of pain elaboration. While the N2/P2 complex is strongly influenced by affective-motivational components of pain, the N1 response, generated by the secondary somatosensory cortex, is considered a pre-perceptive component, linked to the discriminative aspect of pain and not influenced by attention, emotion or intensity of perception [22]. Therefore the N1 lack of habituation observed in our study may represent a marker of abnormal reactivity of the sensory cortex in migraine, probably more constant and reproducible than N2/P2.

This observation is important to define the putative mechanism of action of topiramate in migraine patients. We have shown that this drug is able to specifically modify migraineur’s abnormal behaviour in sensorial processing, confirming previous results obtained using CNV, an event-related cortical evoked potential. In particular, De Tommaso et al. found a correlation between topiramate and levetiracetam efficacy and habituation deficit reduction of CNV potential [17]. It seems therefore that topiramate may act on a general hyperreactivity of the migrainous brain, probably stabilizing cortex excitability. This drug is in fact able to increase the levels of glutamine (GABA precursor) in the cortex [23], to antagonize AMPA and Kainate glutamate receptors from the dorsal root ganglion to the cortex [24,25] and to potentiate GABAergic transmission [18].

In our study topiramate normalized amplitude changes of both LEPs components, which resulted the same as healthy volunteers after therapy (Figures 1 and 2), but its action was more evident on N1 response. The effect of this drug on the early LEPs component may in fact indicate a specific modulation of the sensory-discriminative component of nociception. To our knowledge, this is the first evidence of a specific action of topiramate on different steps of central pain processing in episodic migraine.

Topiramate has previously been shown to modify CNV habituation [17], which is strongly influenced by attention and emotion, and thus one may hypothesize a modulation of this drug on N2/P2 LEPs component. However, different generators and circuits involved in CNV and LEPs could explain this difference. While CNV is a cognitive potential appearing during a reaction time task with a warning and an imperative stimulus, thought to reflect the level of expectation, N2/P2 complex is an evoked potential generated by the emotional response to painful stimulation. Interestingly, a recent study has shown that in nine children, who had a significant reduction in headache frequency after treatment with topiramate, the recovery cycles of the P24 and N30 component of somatosensory evoked potentials were longer after than before therapy. This result suggests that topiramate efficacy in paediatric migraine is probably related to its ability to modify the sensory gating at a somatosensory level, thus restoring cortical excitability [26].

Our findings support this hypothesis, indicating that the main target of topiramate’s induced modulation on pain processing in episodic migraine is the secondary somatosensory cortex. The central role of this area in sensory-discriminative elaboration of pain is well known, although it is also involved in learning, cognition and memory of painful events and in nociceptive modulation through its connection with the thalamus, the prefrontal and insular cortex. Neurotransmitters and receptors involved in these neural circuits are glutamate and oppioids [27]. Topiramate, which modulates glutamate transmission, might thus reduce headache frequency mainly producing functional changes of the complex activity of this cortex. To support this hypothesis, we found a significant correlation between clinical efficacy of topiramate and normalization of N1 habituation pattern.

Moreover, it has recently been shown in mice that nociceptive laser stimulation after systemic administration of morphine generates different effects in somatosensory and anterior cingulate cortices [28]. This finding seems to indicate a separate action of the two areas in the modulation of nociceptive transmission and in their successive elaboration. In this regard, the finding of a modulation of N2/P2 habituation in medication overuse headache after efficacious preventive therapy is quite interesting [14]. In this pathology the anterior cingulated cortex could in fact be particularly involved, as shown by a PET study [29]. Unfortunately, Ferraro et al. did not provide data on the effects of a specific prophylactic agent [14].

Nevertheless, the action of migraine prophylactic drugs cannot be simply related to a single mechanism, as it probably involves the restoring of several functional brain abnormalities and neurotransmitter dysfunctions.

In animal models, for instance, an effect of topiramate on cortical spreading depression and trigeminal activation has been previously reported [19,30]. In addition, a recent paper pointed out a central action of topiramate within the ascending trigeminothalamic pathway via kainate receptors that may probably modulate brain activity. This is particularly interesting, as these receptors are not widely expressed in the brain, but they are certainly found in structures involved in migraine pathogenesis, such as the trigeminal ganglia, the thalamus and the sensory cortex [25].

In the view of individualizing preventive treatment, it could be intriguing to evaluate the specific effects of prophylactic drugs on pathophysiological patterns that characterize migraine patients. In this way the lack of habituation of the sensorial cortex may potentially represent a useful clinical tool. The present study confirms this hypothesis, showing that the clinical efficacy of topiramate is associated with a specific action on the neuronal events predisposing to attack onset.

Finally, the subjective perception of pain after nociceptive stimulation was reduced in patients at baseline respect to controls. Interestingly, it has recently been shown by means of fMRI that migraine patients present an increased antinociceptive activity, which the authors explained as a compensatory reorganization to modulate pain perception [31]. In our study, topiramate normalized patients’ subjective responses, probably through the restoration of a physiological modulation of pain.

In addition, our finding of reduced first block amplitudes in migraine patients respect to controls is in line with several previous findings and suggests a lower pre-activation level of nociceptive cortex [1,32,33]. The habituation deficit that follows the initial pattern of reduced responses is considered as a compensatory phenomenon [34,35]. This peculiar behaviour perhaps generates a continuous pattern of higher brain activation, probably facilitating the onset and persistence of headache. We observed significant changes in initial N1 amplitudes between T0 and T1 recording sessions in migraine patients (Figure 1). Topiramate might thus act on habituation pattern restoring a normal cortical pre-activation level. The same behaviour has been observed in migraineurs after beta-blockers prophylaxis, showing that different drugs, by modulating separate pathways, may act on the same pathophysiological mechanism. This may thus be a crucial dysfunction, probably constituting the background from which migraine attack develops.

## Conclusions

In conclusion, migraine mechanism cannot be reduced to an over-action of neuronal excitation, but to an altered modulation of sensory processing. Topiramate is able to normalize this dysfunction, acting specifically on the sensory-discriminative component of pain elaboration. In the future, it would be interesting to use LEPs recordings to study and compare the mechanism of action of different migraine preventive treatments.

## Competing interests

The authors declare that they have no competing interests.

## Authors’ contributions

LDC, FP, EV and VDP enrolled patients, followed them for clinical aspects and treatment and drafted the manuscript; AB, AT and GC applied LEPs stimulation and processed recording data; AV made the statistical analysis. All authors read and approved the final manuscript.
